# Root-expressed maize lipoxygenase 3 negatively regulates induced systemic resistance to *Colletotrichum graminicola* in shoots

**DOI:** 10.3389/fpls.2013.00510

**Published:** 2013-12-18

**Authors:** Nasie N. Constantino, Fatemeh Mastouri, Ramadhika Damarwinasis, Eli J. Borrego, Maria E. Moran-Diez, Charley M. Kenerley, Xiquan Gao, Michael V. Kolomiets

**Affiliations:** ^1^Department of Plant Pathology and Microbiology, Texas A&M UniversityCollege Station, TX, USA; ^2^State Key Laboratory of Crop Genetics and Germplasm Enhancement, College of Agriculture, Nanjing Agricultural UniversityNanjing, China

**Keywords:** beneficial microorganisms, oxylipin, priming, hydrogen peroxide, root-to-shoot signaling, *Trichoderma*, *Colletotrichum graminicola*, long distance signaling

## Abstract

We have previously reported that disruption of a maize root-expressed 9-lipoxygenase (9-LOX) gene, *ZmLOX3*, results in dramatic increase in resistance to diverse leaf and stalk pathogens. Despite evident economic significance of these findings, the mechanism behind this increased resistance remained elusive. In this study, we found that increased resistance of the *lox3-4* mutants is due to constitutive activation of induced systemic resistance (ISR) signaling. We showed that *ZmLOX3* lacked expression in leaves in response to anthracnose leaf blight pathogen *Colletotrichum graminicola*, but was expressed constitutively in the roots, thus, prompting our hypothesis: the roots of *lox3-4* mutants are the source of increased resistance in leaves. Supporting this hypothesis, treatment of wild-type plants (WT) with xylem sap of *lox3-4* mutant induced resistance to *C. graminicola* to the levels comparable to those observed in *lox3-4* mutant. Moreover, treating mutants with the sap collected from WT plants partially restored the susceptibility to *C. graminicola*. *lox3-4* mutants showed primed defense responses upon infection, which included earlier and greater induction of defense-related PAL and GST genes compared to WT. In addition to the greater expression of the octadecanoid pathway genes, *lox3-4* mutant responded earlier and with a greater accumulation of H_2_O_2_ in response to *C. graminicola* infection or treatment with alamethicin. These findings suggest that *lox3-4* mutants display constitutive ISR-like signaling. In support of this idea, root colonization by *Trichoderma virens* strain GV29-8 induced the same level of disease resistance in WT as the treatment with the mutant sap, but had no additional resistance effect in *lox3-4* mutant. While treatment with *T. virens* GV29 strongly and rapidly suppressed Zm*LOX3* expression in hydroponically grown WT roots, *T. virens* Δsml mutant, which is deficient in ISR induction, was unable to suppress expression of *ZmLOX3*, thus, providing genetic evidence that SM1 function in ISR, at least in part, by suppressing host *ZmLOX3* gene. This study and the genetic tools generated herein will allow the identification of the signals regulating the induction of resistance to aboveground attackers by beneficial soil microorganisms in the future.

## Introduction

Plants have evolved primary and secondary defense strategies to recognize and protect themselves against their enemies, rendering them resistant to the majority of their pests (Balmer et al., 2013b; Fu and Dong, 2013). The primary defensive layer is often built upon constitutively present defensive structures or molecules, such as, thorns, needles, trichomes, cell wall barriers, and preformed antimicrobial compounds (Pieterse et al., 2009; Balmer et al., 2013a). However, many microbes and herbivores are able to breach this primary layer of defense. To overcome the attenuation, host plants utilize a secondary defensive strategy that is often associated with sophisticated mechanisms of induced resistance (Van der Ent et al., 2009a). Such inducible defense is often characterized by increased resistance against a broad-spectrum of potential pests both locally and systemically in the distal parts from the site of primary invasion (Spoel and Dong, 2012; Balmer et al., 2013a; Walters et al., 2013). This mechanism, known as systemic acquired resistance (SAR), is employed by plants to restrict pathogen expansion in systemic tissue through necrosis at the local site upon primary infection, and typically characterized by the increased level of salicylic acid (SA) and activation of SA-responsive genes, such as those encoding pathogenesis-related (PR) proteins (Durrant and Dong, 2004; Vlot et al., 2008).

Another class of inducible defense, known as “induced systemic resistance” (ISR) is activated upon colonization of roots by mutualistic microbes (Choudhary et al., 2007; Pozo and Azcon-Aguilar, 2007; Van Wees et al., 2008; Van der Ent et al., 2009a; Shoresh et al., 2010; Balmer et al., 2013b; Walters et al., 2013). A number of beneficial microorganisms are known to induce ISR in monocots and dicots through ethylene (ET) and jasmonate (JA)-dependent signaling pathways (Van der Ent et al., 2009b). Previous studies have shown that colonization of maize roots by *Trichoderma* spp. triggers systemic resistance to foliar infection by *Colletotrichum graminicola.* Various elicitors of ISR have been isolated from *Trichoderma* spp., and a recent study has shown that a secreted hydrophobin-like elicitor, SM1, plays an essential role in the elicitation of ISR in maize and other crops, as Δ*sm1* mutant lacks the ability to induce ISR (Djonovic et al., 2007).

There are a number of differences between SAR and ISR. JA, ET (Bostock, 2005; Thaler et al., 2012), and volatile organic compounds (VOCs) including green leaf volatiles (GLVs) (Pieterse et al., 2009; Mathys et al., 2012; Weller et al., 2012; Balmer et al., 2013b) are implicated as signals in ISR, while SA and its methyl derivative, along with several other recently identified metabolites might act as the signaling molecules in SAR. It is reported that activation of several PR proteins, e.g., PR1, PR2, and PR5 are not associated with the ISR pathway (Van Wees et al., 1999; Ryu et al., 2004; Ton et al., 2007), and generally ISR is independent of the SA signaling pathway (Pieterse et al., 1996). It has been argued that due to the fitness costs of constitutively expressing ISR or SAR, plants have evolved these inducible mechanisms when encountered with pathogens or insects that break through their first layer of defense (Heil, 2001; Heil and Baldwin, 2002). Thus, a number of negative regulators of these JA- and SA-inducible pathways have likely evolved coordinately (Chini et al., 2007; Hu et al., 2013).upon appropriate stimulation, such us some interaction with pathogens, insects or mutualistic microorganisms, a unique physiological state known as “priming” can be induced. Priming involves subtle regulation of defense related pathways in local and systemic tissues that follow faster and/or greater induction of defense mechanisms upon future encounters (Conrath et al., 2006). Priming protects plants against a broad range of biotic (Conrath et al., 2006) as well as abiotic challenges (Harman et al., 2004; Waller et al., 2005; Baltruschat et al., 2008; Mastouri et al., 2012) and ecological studies have shown that the benefits associated with priming outweighs the cost (van Hulten et al., 2006).

**Figure 1:**
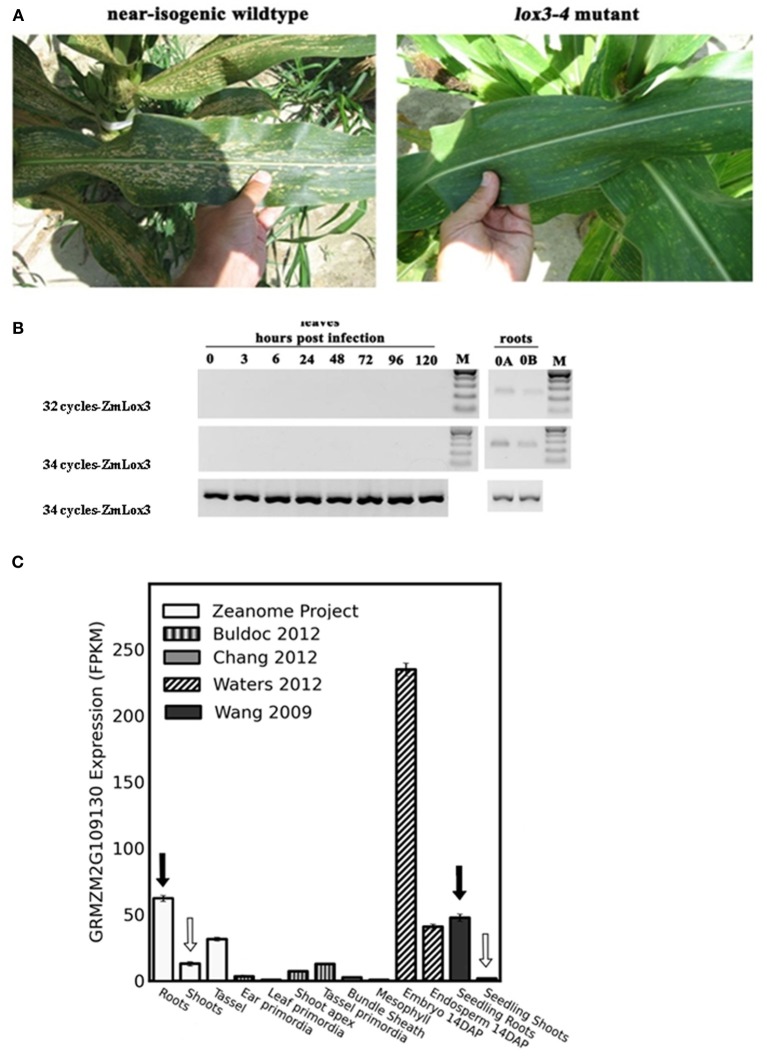
***Colletotrichum graminicola* susceptibility factor, *ZmLOX3*, accumulates preferentially in belowground tissue of maize. (A)** Southern corn leaf blight disease symptoms observed under natural field conditions in 2006, College Station, TX, USA of *lox3-4* mutant and near-isogenic wild-type maize leaves of the FR2128 background. **(B)** Semi-quantitative reverse-transcriptase PCR analysis of *ZmLOX3* transcript accumulation in *C. graminicola* infected maize leaves and in untreated roots at 32 and 34 cycles. *GAPc* at 30 cycles was used as an internal control to normalize cDNA template concentrations. **(C)** *ZmLox3* gene expression from several RNA-seq sources assembled through qTeller. Highlight is expression in leaves (white arrows) comparable with roots (black arrows).

In our previous studies (Gao et al., 2007; Isakeit et al., 2007), we found that disruption of a 9-lipoxygense (9-LOX) of maize, *ZmLOX3*, resulted in a remarkable increase in resistance to multiple seed, root, leaf, and stem fungal pathogens, including seed contaminating mycotoxin-producing *Fusarium verticillioides*, southern leaf blight caused by *Cochliobolus heterostrophus* (Figure 1A) and stalk rots caused by both *F. verticillioides* and *C. graminicola*, respectively. Although this resistance is effective against a broad range of pathogens in diverse organs, but regardless of infection, *ZmLOX3* transcripts are hardly detectable in the aboveground organs (Gao et al., 2007). Moreover, changes of a set of defense-related genes, including JA/SA/ET-responsive and biosynthetic genes, as well as endogenous levels of JA, SA, and ET were specifically elevated in the roots, but not in the leaves of *lox3-4* mutants (Gao et al., 2007). This prompted our major hypothesis tested in this study: the abnormal constitutive activation of all three major defense pathways in the roots of *lox3-4* mutant are responsible for the production of yet to be identified signal(s) responsible for constitutively active systemic resistance in all aboveground organs of maize against distantly-related pathogens.

Anthracnose leaf blight, one of the most devastating diseases of maize caused by *C. graminicola*, was employed as the model disease for this study. *C. graminicola* is a hemibiotroph, with a well-defined transition from biotrophic to necrotrophic stage in maize. *C. graminicola* induces tremendous changes in the transcriptome of the host plant at the site of inoculation as well as in systemic tissue (Vargas et al., 2012; Balmer et al., 2013b). Defense related genes are upregulated in both local and systemic tissues of the host as early as 12 h post inoculation (hpi) (Vargas et al., 2012; Balmer et al., 2013b), and continue to increase to peak at ~60 hpi when the pathogen switches to the necrotrophic stage of its life cycle. These findings indicate that the pathogen does not suppress the common defense mechanisms of the host. Instead, it has been suggested that *C. graminicola* switches lifestyles to evade the increasing host defense machinery against biotrophic pathogens (Vargas et al., 2012). Defense, therefore, seems life-style dependent on a subset of distinct biochemical processes. Maize plants accumulate H_2_O_2_ in cells adjacent to the fungal hyphae, suggesting its involvement in defense against *C. graminicola* (Vargas et al., 2012) and in promoting cell wall lignification around the site of pathogen penetration (Torres and Dangl, 2005). H_2_O_2_ accumulation is suggested to limit growth of hemibiotrophic fungi such as *Septoria tritici* during both biotrophic and necrotrophic stages (Shetty et al., 2007), while the pathogenicity of the *Magnaporthe oryzae* is shown to be directly related to the ability to scavenge and detoxify ROS (Huang et al., 2011).

To test our hypothesis and probe the molecular mechanism underlying the systemic resistance observed in *lox3-4* mutant, as well as to identify the potential molecules responsible for ISR-like activity, we performed a series of experiments using the xylem sap collected from *lox3-4* mutant roots. We found that WT plants treated with the sap of *lox3-4* mutant showed enhanced resistance to *C. graminicola*, which was almost identical to the levels of ISR induced in WT maize when colonized by the beneficial fungus *T. virens*, and the resistance levels observed in the untreated *lox3-4* mutant. However, no such ISR-like activity was observed in either WT or *lox3-4* mutant in response to xylem exudates from WT, suggesting the constitutive nature of ISR signaling in the *lox3-4* mutant. The results of comparative gene expression revealed that ISR-inducing strain of *T. virens* down-regulates *ZmLOX3* in host roots. In contrast, the mutant strain, in which ISR-inducing SM1 protein was deleted, was not able to suppress expression of *ZmLOX3*. These results explain for the first time the molecular mechanism of the remarkable resistance of *lox3-4* mutant to multiple pathogens, and implicate the *ZmLOX3* gene as a target for SM1-mediated ISR elicitation.

## Methods

### Plant and fungal materials

The *lox3-4* mutants were identified using a reverse genetics resource generated by *Mu*-transposable element-insertional mutagenesis as described previously (Gao et al., 2008). Both the mutant and wild type plants (WT) are near-isogenic lines (NILs) at the backcross 7 (BC7) genetic stage in the B73 background. In addition to B73, the *lox3-4* mutant allele has been backcrossed into FR2128 four times (BC_4_), and the increased resistance of *lox3-4* in this genetic background is presented in Figure 1A. For the remaining experiments, the mutant in the B73 genetic background was used so that it can be directly compared to the B73 line (hereafter designated WT). The mutant and WT seeds were grown at approximately 25–28°C in commercial soil (Metro-Mix 366; Scotts-Sierra Horticultural Products, Marysville, OH, U.S.A.) under 14 h of daylight with 150 μmol m^−2^ s^−1^ (Quantum Meter; Apogee Instruments, Logan, UT, U.S.A.). The seedlings were grown in long conical tubes (20.5 by 4 cm) for 2–3 weeks until they had three fully expanded leaves (V3 developmental stage). To study the effect of root colonization by *T. virens* on *C. graminicola* disease development, WT and *lox3-4* seed were treated with *T. virens* GV29-8 strain according to Djonovic et al. (2007) and sown in conical pots (15 by 3 cm) filled with steam-sterilized coarse sand/peat mix (6:1, vol/vol).

To study the effects of *T. virens* colonization of roots, we used a hydroponic system as described previously (Djonovic et al., 2007). This system was chosen to prevent contamination with microbes and allow root sampling with minimal mechanical damage (Supplementary Figure 1A). Briefly, disinfected seeds were germinated over 2 days on LDB medium and microbe-free germinated seedlings were transferred to hydroponic vessels containing half-strength Murashige and Skoog (1/2 MS) medium amended with Gamborg's vitamins (pH = 5.6) (Sigma-Aldrich, St. Louis, MO, U.S.A). Strains of *T. virens* were grown and applied to the hydroponic media as described by Djonovic et al. (2007) 24 h after the seedling transfer.

*C. graminicola* strain 1.001 was cultured on potato dextrose agar, and the conidial suspension for infections was prepared and infection performed as described by (Gao et al., 2007).

### Xylem sap collection and application to seedling stems

WT and *lox3-4* plants were grown to V4 developmental stage (4th leaf fully developed) in a greenhouse under natural light (during months of April until July). One day before sap collection, the plants were transferred to lighted shelves (150 μmol m^−2^ s^−1^ (Quantum Meter; Apogee Instruments, Logan, UT, U.S.A.) with 14 h of daylight and were immediately watered. 24 h later, plants were re-watered until the soil was saturated, and then plants were carefully decapitated with a sharp scalpel above the first leaf (Supplemental Figure 1B). The first droplet of sap was discarded and subsequent droplets were collected for 8 h and stored on ice. The plants were periodically recut when cut site sealed, the next droplets were discarded as above. Sap collected from plants of the same genotype was mixed thoroughly before freezing in liquid N_2_ and stored at -80°C until needed for the experiments. B73 plants were grown to the V4 stage to match the developmental stage of the collected sap. WT and *lox3*-*4* sap were thawed and diluted 1:1 with sterile distilled water to reduce viscosity. Plants were placed horizontally in a sterilized tray and two 1 cm long incisions were made between the first and second leaves (Supplemental Figure 1C). The incisions were made halfway through the depth of the stem so as not to puncture the backside, and then 50 μl of diluted sap was added to each incision. Masking tape was used to seal the wound sites and to ensure that the sap did not leak out.

### RNA extraction, semi-Q-RT-PCR and Q-RT-PCR analysis

Leaves from the mutant and WT plants were harvested at designated times after inoculation and were immediately frozen in liquid N_2_, and stored at −80°C. Roots of hydroponically grown plants were harvested at designated incubation intervals, excised with a sharp scalpel, immediately frozen in liquid N_2_, and stored at −80°C. Total RNA was extracted from tissues using TRI Reagent (Molecular Research Center, Inc., Cincinnati, OH, U.S.A) and treated with DNase according to the manufacturer's instructions (RNase-free DNase kit, Qiagen, Valencia, CA, U.S.A). For semi-quantitative PCR (semi-Q-RT-PCR) experiments, 1 μg of RNA was used for cDNA synthesis as described previously (Gao et al., 2007) with GAPc used as a reference gene.

For quantitative reverse transcription-PCR (Q-RT-PCR) assays, a one-step qPCR procedure was performed using Thermo Scientific Verso One-Step RT-qPCR Kits (Thermo Scientific, Waltha, MA, U.S.A). Reactions were optimized for RNA and primer concentrations with each 10 μ l reaction consisting of 40 ng of DNase-free RNA and 200 nM primers. Q-RT-PCR analysis was performed with an Applied Biosystem StepOne Plus Real-Time PCR instrument. Primers used in this study are described in Supplementary Table 1. The PCR program consisted of a 15 min cDNA synthesis step at 50°C, followed by polymerase activation step of 15 min at 95°C, followed by 40 cycles of 15 s at 95°C, 30 s at 56°C, and 30 s at 72°C followed by a melt curve analysis. Primers were designed using Primer3Plus software in accordance with the criteria required for quantitative PCR primer design (Udvardi et al., 2008). The specificity of primers, lack of primer-dimer formation, and the absence of contaminating genomic DNA was verified, respectively, using amplicon dissociation curves, PCR in the absence of cDNA, and by PCR analysis of RNA samples before reverse transcription. The amplification efficiency of primers was calculated using LinReg (11.0) was ≥ 80%. Initially, the *GAPc* and *Cullin* genes were tested for reference gene suitability, and *Csullin* was chosen based upon its more stable expression across samples. Expression levels were normalized using *Cullin* as a reference gene, and relative expressions of genes compared to control (time 0) were calculated using the method of Ruijter et al. (2009).

### Bioinformatics

*ZmLOX3* RNAseq expression data was compiled from publically available gene expression data through qteller (http://qteller.com/qteller3/bar_chart.php?name=GRMZM2G109130

### Hydrogen peroxide detection in response to infection and treatment of maize leaf with alamethicin

The leaf segments (approximately 5 cm length) of *lox3-4* mutant and WT plants at V4-stage were treated with alamethicin (ALA, Sigma-Aldrich, St Louis, MO, U.S.A) at 10 μg/mL by applying 10 μl of solution to the midrib vain of the leaf segment and incubating for 48 h. H_2_O_2_ production in WT and *lox3-4* mutants upon ALA treatment was examined according to the DAB staining method (Gao et al., 2008) with modifications. Briefly, leaves were excised and subsequently immersed in DAB (1mg/ml; pH 3.8; 3, 3′-diaminobenzidine, Sigma-Aldrich, St Louis, MO, U.S.A) solution with low vacuum pressure for 30 min, followed by an overnight incubation at room temperature in the dark. The stained leaves were fixed and cleared in alcoholic lactophenol (95% ethanol: lactic acid: phenol = 2: 1: 1) at 65°C, rinsed once with 50% ethanol, and twice with H_2_O. The destained leaves were stored in 50% glycerol or subjected to microscopic observation.

### Nitric oxide (NO) detection in the leaves

To visualize NO production, the leaf segments were excised from 3-week-old *lox3-4* mutant and WT seedlings at the V4 stage and injected with 1ug/mL ALA on the middle site of main vein of leaf. NO production was examined by diaminofluorescein (DAF) as described by Delledonne et al. (1998).

### Distribution of materials

Novel materials described in this publication may be available for non-commercial research purposes upon acceptance and signing of a material transfer agreement. In some cases such materials may contain or be derived from materials obtained from a third party. In such cases, distribution of material will be subject to the requisite permission from any third-party owners, licensors or controllers of all or parts of the material. Obtaining any permission will be the sole responsibility of the requestor.

## Results

### *ZmLOX3* is preferentially expressed in roots but not in infected leaves

Our previous studies showed that elimination of the *ZmLOX3* gene in B73 inbred line background conferred increased resistance to *C. graminicola* infection in leaves (Gao et al., 2007). Since that study, we have created near-isogenic WT and mutant lines in FR2128 genetic background. As shown in Figure 1A, similar to what was observed in the B73 background in the previous study, disruption of *ZmLOX3* in the FR2128 genetic background resulted in easily visible and strong reduction of disease severity to southern corn leaf blight under natural field conditions. Surprisingly, no *ZmLOX3* transcripts could be detected by semi-Q-RT-PCR analyses even at an unusually high number of PCR cycles during the entire time-course of infection by *C. graminicola* (Figure 1B). Importantly, the time-course tested was extended for 96 h, a time point by which most infected tissue is macerated by the fungus. As a positive control for RT-PCR analyses, *ZmLOX3* transcripts accumulated in the roots. A survey of *ZmLOX3* gene expression from several RNAseq sources assembled through qTeller revealed that *ZmLOX3* is preferentially expressed in maize roots and shows low expression in seedling shoots (Figure 1C). These results prompted a hypothesis that disruption of *ZmLOX3*, which is normally expressed in roots, results in a constitutive production of a systemic signal(s) that renders the entire plant, including distal above-ground tissues, resistant to *C. graminicola* and other pathogens (Gao et al., 2007).

### Expression of defense related genes in *lox3-4* mutant upon *C. graminicola* infection

To explore the molecular mechanism of the enhanced resistance in *lox3-4* mutants, we examined the expression of a set of defense-related genes in the leaves of both the mutant and near-isogenic WT upon infection with *C. graminicola*. Genes used for this study were pathogenesis related protein 1 (*PR1*), hydroperoxide lyase (*HPL*), phenylalanine ammonia lyase (*PAL1*), glutathione S-transferase (*GST*), and maize proteinase inhibitor (*MPI*). GAPc was used as the reference gene.

**Figure 2:**
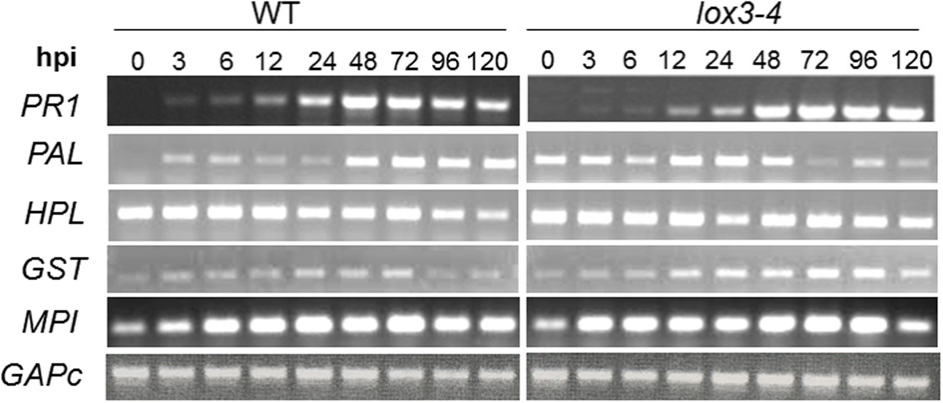
**Expression of defense-related genes in leaves of *lox3-4* mutant vs B73 NIL (WT) in response to *C. graminicola* infection.** Approximately 3-week-old *lox3-4* mutant and WT seedlings at 4 true leaf stage were infected with *C. graminicola* at 1 × 10^6^ spores.ml^−1^.mlnicolaola with with at 4 tTrizol reagent and cDNA synthesized with Ambion 1st strand cDNA synthesis kit. PCR was conducted using standard procedures and the expression of GAPc was used as an internal control. *HPL*, hydroperoxide lyase; *PAL*, phenylalanine ammonia lyase; *GST*, glutathione S-transferase; *MPI*, maize proteinase inhibitor; *PR1*, pathogenesis related protein 1; *GAPc*, glyceraldehyde-3-phosphate dehydrogenase.

Interestingly, we did not detect any difference in the level of *PR1*, the gene most commonly used as SAR molecular marker, in leaves of *lox3-4* or WT plants in response to *C. graminicola*, indicating that the observed resistance of the mutant may not be due to SAR. *PAL1* and *MPI* were induced earlier in the mutant leaves in response to inoculation with *C. graminicola* and *GST* was induced earlier and stronger in mutant than in WT (Figure 2). *PAL1* showed higher expression in the leaves of the mutant even at time 0, and was further enhanced in *lox3-4* mutants compared to WT. The striking similarity of these results with those previously reported in maize colonized with *T. virens* (Djonovic et al., 2007) prompted us to postulate that *lox3-4* mutant plants may constitutively express an ISR-like phenomenon that induces wide-spectrum systemic resistance to foliar leaf blight and other diseases reported previously (Gao et al., 2007).

### Xylem sap extracted from *lox3-4* mutant increased resistance of wt plants to *C. graminicola*

**Figure 3:**
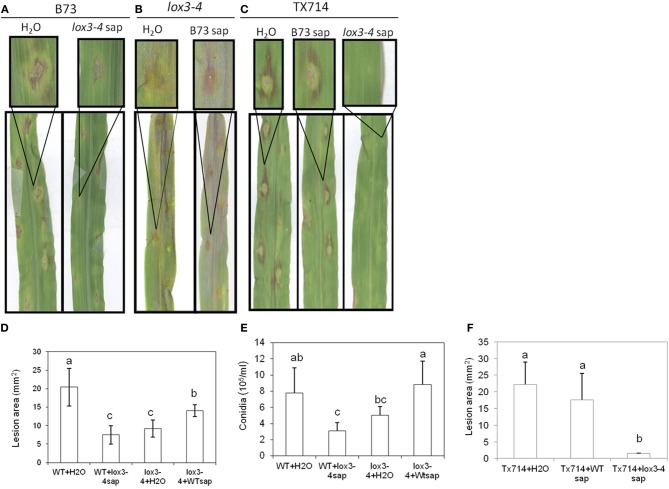
**Effect of xylem sap from *lox3-4* mutant and B73 NIL plants on the pathogenicity of *C. graminicola* infected on the leaves.** The xylem sap were collected from approximately 3-week-old *lox3-4* mutant and B73 seedlings at 4 true leaf stage by cutting the stem from the position above the soil. Twenty microliter of sap per plant was injected into the wounding site on the stem of *lox3-4* mutant and B73 or TX714 NIL, respectively, subsequently followed by the infection with *C. graminicola* at 1 × 10^6^ spores/ml. The disease symptoms were recorded and photographed at 3 days post infection on **(A)** B73 plants treated with H2O or *lox3-4* xylem sap, **(B)** *lox3-4* plants treated with H2O or B73 sap, and **(C)**TX714 plants treated with H2O, B73 sap, or *lox3-4* xylem sap. The insert panels show the close-up of representative lesions showing on the corresponding leaf. The **(D)** lesion size and **(E)** number of conidia on *lox3-4* and B73 leaves infected with *C. graminicola* upon treatment H2O, and *lox3-4* or B73 sap and **(F)** lesion size on TX714 leaves infected with *C. graminicola* upon treatment H2O, and *lox3-4* or B73 sap. The data are shown as mean ± SE (*n* > 15 for lesion size and *n* = 3 for conidia number experiments, respectively). The different letters above the bar indicate the significant difference between different treatments analyzed by SPSS software One-Way ANOVA (*p* < 0.05).

To address whether the induced resistance to *C. graminicola* in the aboveground organs of *lox3-4* mutant resulted from constitutive production of long-distance signal(s) by its roots, xylem sap from WT and the *lox3-4* mutant seedlings were collected and applied to *lox3-4* and WT plants and followed by inoculation with *C. graminicola*. Consistent with our previous findings (Gao et al., 2007), *lox3-4* mutant showed increased resistance to *C. graminicola* compared to WT (Figures 3A,B,D,E). Application of xylem sap collected from WT plants on *lox3-4* mutant seedlings compromised the resistance level observed in *lox3-4* mutants as evidenced by greater lesion area and increased conidial production (Figures 3B,D,E). Remarkably, application of *lox3-4* mutant sap to WT plants significantly increased resistance as compared with that of WT supplemented with either H_2_O or WT sap (Figures 3A,D,E), evident by significant reduction in lesion size and conidial formation (Figures 3D,E). In addition to the mutant sap-conferred resistance in B73, the application of *lox3-4* sap resulted in even more dramatic decrease of disease symptoms in leaves of Tx714 inbred line (Figures 3C,F). These results suggest that the long-distance signal(s) in xylem sap of *lox3-4* is capable of inducing disease resistance in diverse genetic backgrounds. Taken together, these results strongly support the hypothesis that *lox3-4* mutant roots constitutively produce an ISR-like long-distance signal that is transported to above-ground organs via xylem sap.

**Figure 4:**
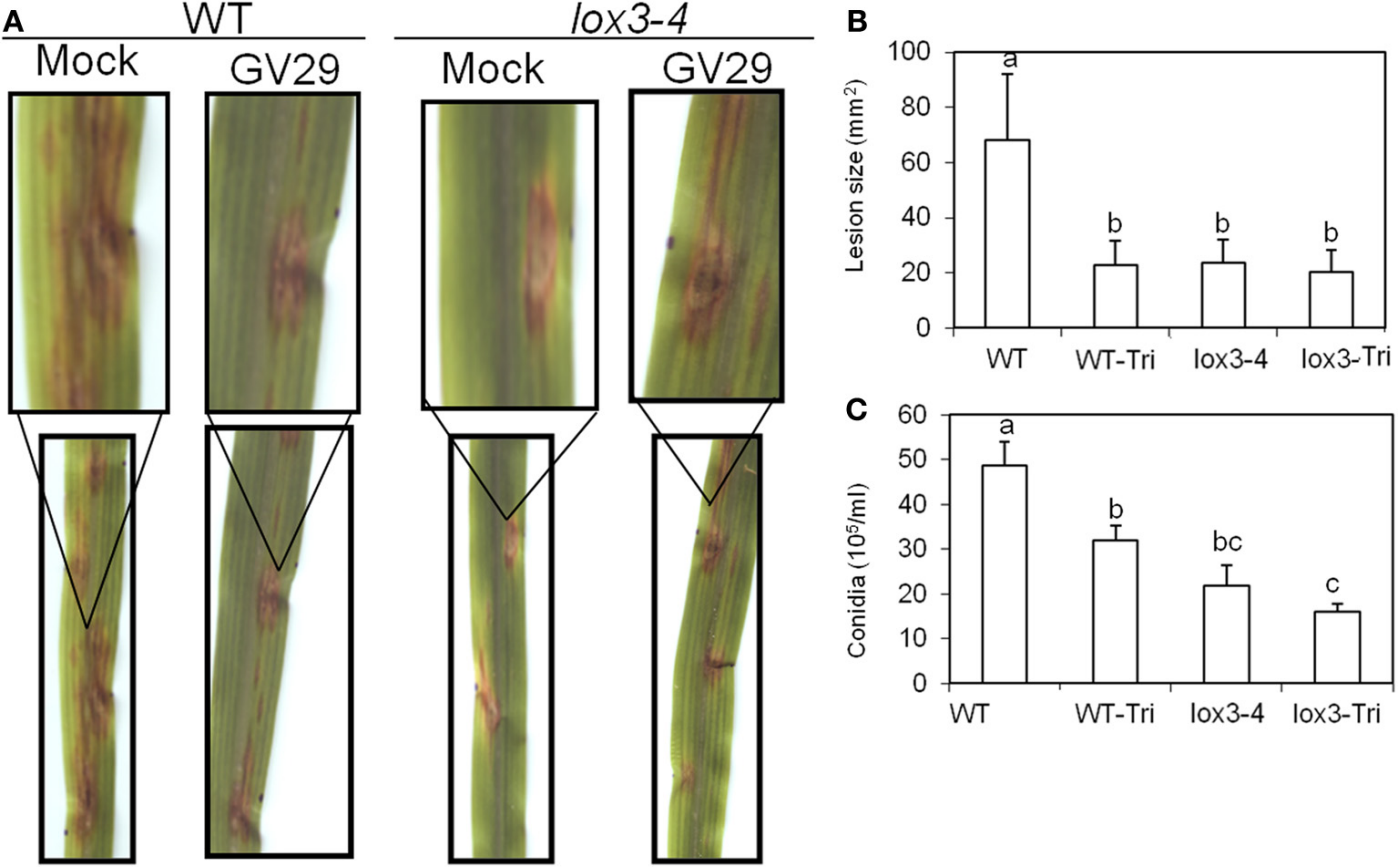
**Effect of colonization of roots of *lox3-4* mutant and B73 plants on the pathogenicity of *C. graminicola* infected on the leaves.** Approximately 3-week-old *lox3-4* mutant and B73 seedlings at 4 true leaf stage were treated with *T. virens* in the roots. Then the leaves of treated plants were infected with 10 μ l of *C. graminicola* conidial suspension at 1 × 10^6^ spores/ml at 3 days post treatment. **(A)** The disease symptoms were recorded and photographed at 3 days post infection. The insert panels show the close-up of representative lesions showing on the corresponding leaf. **(B)** The lesion size on *lox3-4* and B73 leaves infected with *C. graminicola* upon treatment with or without *T. virens*. The data are shown as mean ± SE (*n* > 15) of lesions examined. **(C)** The conidia number of *C. graminicola* from the infected leaves of *lox3-4* mutant and WT leaf segment upon treatment with or without *T. virens*. The data are shown as mean ± SE (*n* = 3) of leaves examined. The different letters above the bar indicate the significant difference between different treatments analyzed by SPSS software One-Way ANOVA (*p* < 0.05).

Interestingly, the level of reduction of lesion size triggered by *lox3-4* xylem sap in WT was identical to treatment of WT plants with *T. virens* (Figures 4A,B,C). Furthermore, root colonization by *T. virens* did not enhance resistance of *lox3-4* mutants, indicating that *lox3-4* mutants constitutively express an ISR-like signal transported through xylem sap that induces resistance in the leaves.

### *ZmLOX3* is down-regulated by *T. virens*

**Figure 5:**
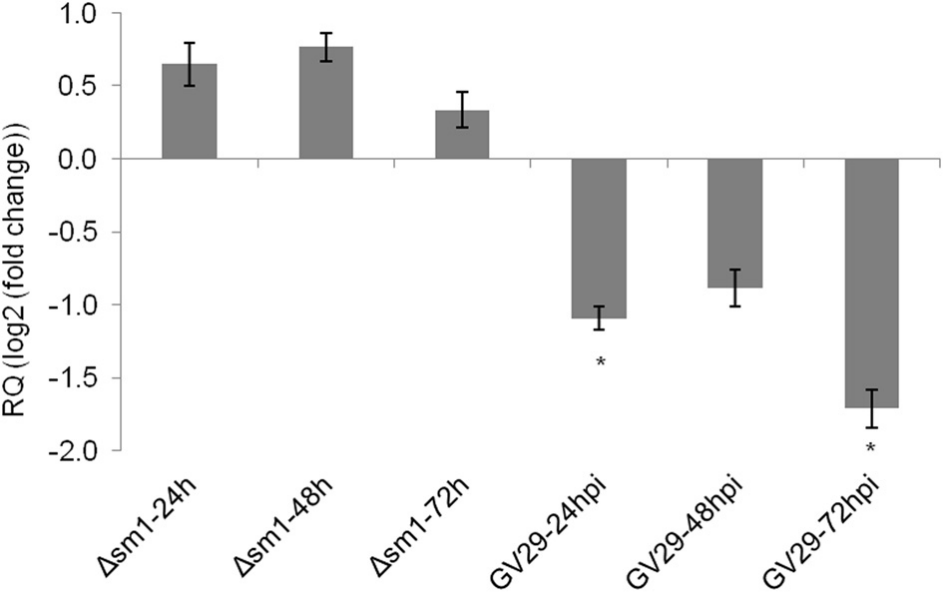
**Relative expression of ZmLOX3 in response to root colonization by T. virens strain GV-29-8 and Δ sm1, a mutant that is unable to induce ISR in maize.** Two days old disease free seedlings were transferred to hydroponic media with half-strength Murashige and Skooge media and were inoculated with the hyphae of the fungus. At each time point, three biological replicates, each consisting of three plants were sampled. The experiment was repeated twice and results were pooled and analyzed together. The data are shown as mean log_2_ (fold change) ± SE and only those means those sample with statistically significant change in gene expression (α = 5%) are designated with an asterisk.

The results presented above provide strong evidence that *ZmLOX3* acts as a potent negative regulator of ISR. If this hypothesis is true, then we reasoned that ISR-inducing microorganisms must suppress this gene to facilitate ISR activation. To test this hypothesis, we compared *ZmLOX3* expression in roots of WT maize in response to colonization by ISR-inducing *T. virens* GV29 and the Δ*sm1* mutant strain. In our previous study, we demonstrated that this mutant strain is incapable of inducing ISR against *C. graminicola* in maize (Djonovic et al., 2007). Interestingly, root colonization by GV29 suppressed *ZmLOX3* expression in the roots as early as 24 hpi in the hydroponic system, a time point at which there is no observable root colonization occurring, indicating that the secretion of SM1 from hyphae of *T. virens* has an effect on *ZmLOX3* expression in the hydroponic system used (Figure 5). The expression of this gene remained suppressed compared to control at 48 and 72 hpi when fungal colonization of roots typically occurs, although the difference with control was only significant at 24 and 72 hpi. Importantly, no change in the expression of *ZmLOX3* was detected in maize roots colonized by Δ*sm1* (Figure 5) suggesting that *ZmLOX3* is one of the target genes by which SM1 induces ISR.

### *lox3-4* plants respond to *C. graminicola* or alamethicin treatment by increased production of ROS and NO

**Figure 6:**
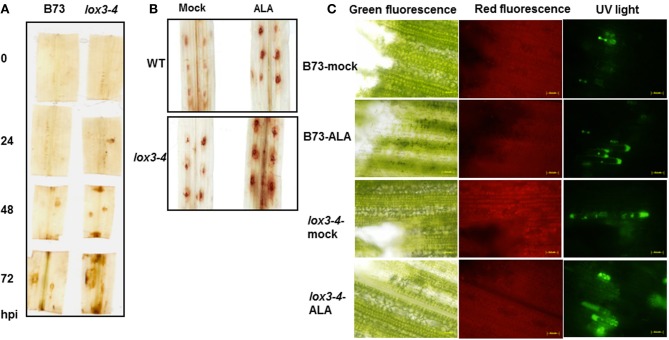
**Effect of (A) *C. graminicola* and **(B)** alamethincin (ALA) elicitor on accumulation of H2O2 or **(C)** Nitric Oxide in *lox3-4* mutant and B73 leaves.** Leaf segment from *lox3-4* mutant and B73 **(A)** inoculated with *C. graminicola* or **(B)** treated with ALA for 24 h were stained according to DAB staining procedure to elucidate the ROS accumulation. **(C)** Nitric oxide accumulation was visualized by DAF fluorecence 4 h after ALA treatment.

To understand the biochemical mechanisms behind increased resistance observed in *lox3-4* mutants, we measured accumulation of H_2_O_2_in leaves of WT and *lox3-4* mutants in response to *C. graminicola* infection. Noticeable change was observed in hydrogen peroxide (H_2_O_2_) accumulation. ROS produced by maize is implicated in defense against *C. graminicola* (Vargas et al., 2012). Using DAB staining, we observed that both WT and *lox3-4* mutants accumulate hydrogen peroxide around the site of *C. graminicola* inoculation. However, *lox3-4* mutants tend to respond faster and accumulate higher hydrogen peroxide levels compared to WT (Figure 6A), which may limit the duration of biotrophic stage of the fungal life cycle during disease development.

To further test if the *lox3-4* mutant is primed for faster ROS accumulation in response to general defense elicitor and not specifically by *C. graminicola*, we tested if such a primed response occured upon treatment with the elicitor alamethicin. Treatment with ALA only slightly increased accumulation of ROS in WT leaf segments, whereas much greater ROS production was detected in ALA-treated *lox3-4* mutant (Figure 6B). Similarly, ALA triggers more NO accumulation in *lox3-4* mutant leaf compared to WT (Figure 6C). ALA increases membrane permeability, a hallmark of lipid peroxidation and hypersensitive response (HR). These results indicate that defense responses were expressed earlier and to a greater extent in the *lox3-4* mutant plants compared to WT, consistent with the previous reported observations of boosted responses associated to a “primed” state in the ISR- expressing plants.

### Xylem sap from *lox3-4* mutant altered response of wt leaves to *C. graminicola* at molecular level

The fourth leaf of WT plants treated with xylem sap extracted from either WT or *lox3-4* mutants were inoculated with *C. graminicola.* Samples were taken at time 0 (the control) and designated times after inoculation to analyze changes in expression of genes involved in basal defense, JA, and other oxylipins biosynthesis. The gene expression analysis was performed using Q-RT-PCR, except for *ZmLOX1*, *ZmLOX2*, *ZmLOX5*, and *OPR7* for which we used semi-Q-RT-PCR. The relative gene expression was determined as fold change compared to the control (time 0).

The relative expression of *PR1* and *PR5* were similar in both treatments, but at 48 hpi leaves of plants treated with *lox3-4* sap showed higher relative expression of both genes than those treated with WT sap. *PAL1* relative expression increased only in leaves of plants treated with *lox3-4* sap at 24 hpi.

The relative expression of genes involved in JA biosynthesis pathway, allene oxide cyclase (*AOC*), 12-oxophytodienoate reductase 7 and 8 (*OPR7* and *OPR8*), were enhanced at earlier time points after inoculation with *C. graminicola* in plants treated with *lox3-4* mutant sap. However, their relative expression levels were reduced or remained unchanged when treated with WT sap.

*ZmLOX5* and *ZmLOX10* were shown to be required for normal colonization of leaves and stalks by *C. graminicola* in maize (Christensen, 2009; Park, 2011). Interestingly, we found that both genes were suppressed in maize leaves treated with sap from *lox3-4* mutants. *ZmLOX5* was not suppressed in leaves of plants treated with WT sap and *ZmLOX10* was actually induced at 12 hpi in these leaves. The expression of *ZmLOX9*, another 13-lipoxygenase that is likely involved in JA biosynthesis (Christensen et al., 2013) was enhanced in leaves of plants treated with *lox3-4* sap at 6 hpi, but not in those treated with WT sap.

## Discussion

Oxylipins, an important class of fatty acid-derived signaling molecules, are implicated in plant defense responses to pathogens, insect herbivory, and abiotic stresses. However, we have previously shown that 9-oxylipins, produced by a maize 9-lipoxygenase, *ZmLOX3*, facilitate pathogenicity of several root, stem, leaf, and seed fungal pathogens (Gao et al., 2007; Isakeit et al., 2007). Maize mutant plants unable to express this gene were shown to gain a remarkable level of increased resistance to a number of diverse fungal pathogens; however, the mechanism of such broad-spectrum resistance was not clear at the time of those publications. Gao et al. (2008) showed that disrupting *ZmLOX3* changes expression of other defense-related genes including other 9- and 13-LOXes and ACC oxidase in root of unstressed and disease-free maize tissue. As a result of this inappropriate defense activation, *lox3-4* mutants are delayed in germination and development of below- and above-ground organs and premature senescence (Gao et al., 2008).

This report provides compelling evidence that the disruption of the *ZmLOX3* gene, normally expressed in roots, results in constitutive production of an ISR-inducing root-to-shoot signal that renders distal tissues resistant to a number of plant diseases. Greater foliage resistance of mutant plants to *C. graminicola* and the potent ability of the mutant xylem sap to increase this resistance in WT plants, clearly indicate that xylem sap of *lox3-4* plants contains a long-distance ISR-inducing signal synthesized in the mutant root. The identity of this signal(s) is currently unknown due to the lack of appropriate analytical and genetic tools. However, we are in the process of creating several combinations of double and triple mutants with JA, green leaf volatile, and ethylene producing enzymes in the background of *lox3-4* mutant to disrupt the constitutive signaling and to enable the identification of the candidate signal(s).

**Figure 7:**
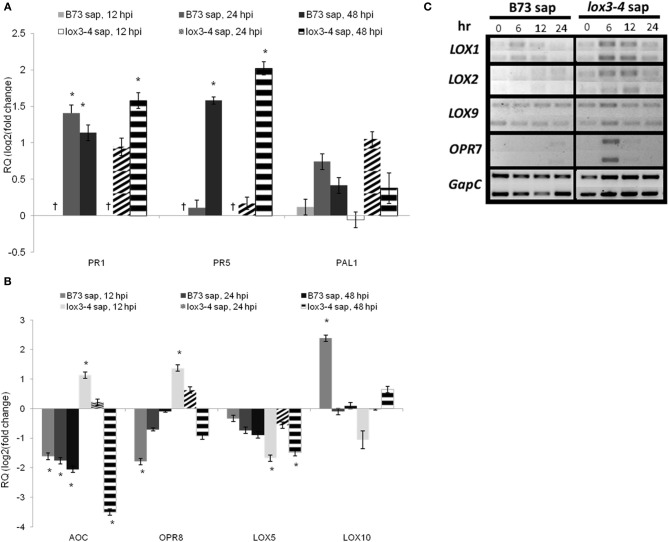
**Effect of B73 and *lox*3-4 sap on the expression of defense genes in B73 maize inoculated with *C. graminicola*. (A)** relative expression of SAR-related genes *PR1* (pathogenesis related 1), *PR5* (pathogenesis related 5), and *PAL1* (phennyl alanine amonia lyase), **(B)** relative expression of genes involved in octadecanoid pathway, AOC (allene oxide synthase), OPR8 (12-oxo-phytodienoic acid 8), LOX5, and LOX10 (lipoxygenase 5 and 10, respectively) and **(C)** semi-Q-RT-PCR analysis of expression of LOX1, LOX2, LOX9, and OPR7. B73 maize plants at V4 stage were treated with xylem sap from B73 or lox3-4 plants (as described in materials and methods) and were inoculated with C. graminicola 3 h later. Control sample was taken prior to sap treatment and the rest of the samples were taken at designated times after inoculation. For **(A)** and **(B)** quantitative real-time PCR (Q-RT-PCR) was used to examine the gene expression and Cullin was used as reference gene. **(C)** Wherever efficiency of q-RT-PCR reaction was bellow 80% semi-Q-RT-PCR analysis was used to study the gene expression and GAPc was used as internal reference. The data are presented as mean ± SE. The ^†^represents the sample that no amplification was detected for the gene expression. The sample with statistically significant change in gene expression (α = 5%) are designated with an asterisk.

**Figure 8:**
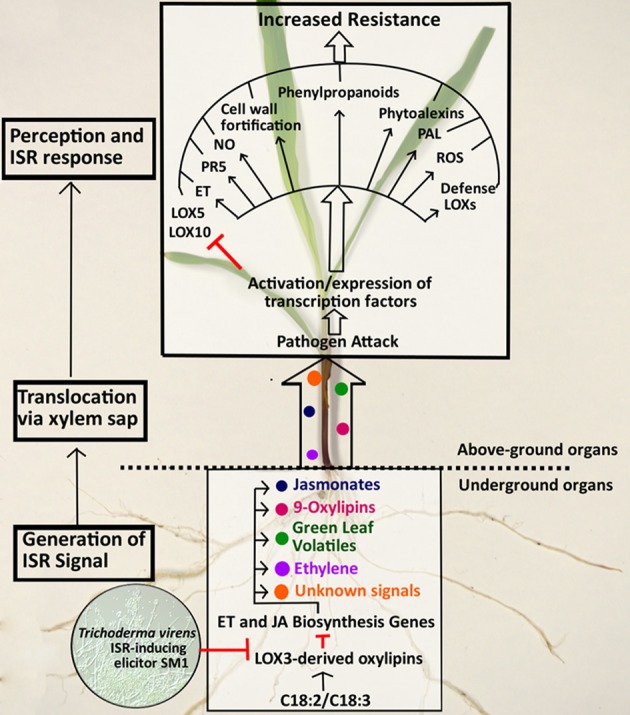
**Working model of *ZmLOX*3 involvement in suppression of ISR Responses.** In unperturbed maize roots, LOX3-derived oxylipins down-regulate defense-related genes and maintain levels of the defensive and developmental hormones, jasmonate (JA) and ethylene (ET) at healthy levels required for normal seed germination, plant growth, and normal life span (Gao et al., 2008). Upon colonization of roots, *Trichoderma virens* produces small molecules that possesses signaling activity and modulate gene expression in the roots to allow for mutualistic interactions with the host. One of such signal effecting expression of the host genes is a secreted proteinaceous elicitor, SM1 (Djonovic et al., 2007). One of the functions of SM1-mediated signaling is the down-regulation of expression of the host gene, *ZmLOX3*, which, in turn, results in de-repression of several defense and signaling host molecules including 9/13-LOX products (such as JA, traumatin, green leaf volatiles, or other oxylipins derived either from C18:3 or C18:2 polyunsaturated fatty acids) and ET. Some of these metabolites, their derivatives or their precursors are transported via xylem into aboveground organs where they signal changes in transcription of a number of defense-related genes including, upregulation of pathogenesis-related genes (e.g., PR5), phenylalanine ammonia lyase (PAL), several defense related LOXs such as LOX1, LOX2, LOX9, and other genes required for biosynthesis of JA, ROS, NO, and cell-wall fortification (e.g., lignin), phytoalexins, and other defense-related metabolites. In addition to up-regulation of defense genes, the host susceptibility genes that promote colonization of tissues by diverse pathogens are down-regulated. This includes LOX10 and LOX5 genes shown to promote disease (Christensen, 2009; Park, 2011). Collectively, these reprogramming in metabolome and transcriptome results in heightened defense status known as ISR in systemic tissues leading to increased resistance upon pathogen attack.

Comparative gene expression showed that the treatment of WT plants with *lox3-4* sap resulted in a significant change of the transcript accumulation pattern in these plants when infected with *C. graminicola* compared to WT plants treated with WT sap (Figure 7). Specifically, WT plants treated with *lox3-4* sap responded to the inoculation with faster and stronger induction of genes involved in JA biosynthesis, i.e., 13- or 9/13-*LOXs*, *LOX1*, *LOX2*, *LOX9*, *AOC*, *OPR7*, and *OPR8*. Other defense-related genes including *PR1*, *PR5*, and *PAL1* were also differentially expressed in *lox3-4-*sap treated WT plants. *PAL1* was only induced in WT plants treated with *lox3-4* sap at 24 hpi, and *PR1* and *PR5* were expressed at higher level in WT plants treated with the *lox3-4* sap. Interestingly, *PAL1* gene was constitutively expressed in uninfected *lox3-4* mutants as well as in infected plants throughout the entire time course examined, but was induced later and to lower levels in WT plants infected with the pathogen (Figure 2). PAL enzymes are involved in the biosynthesis of SA and other phenolic compounds, which are implicated in induction of defense in systemic tissue, and are required for cell wall strengthening via lignification processes (Mauch-Mani and Slusarenko, 1996; Chen et al., 2009) Therefore, we speculate that these PAL-derived defense metabolites may be one potential mechanism behind increased resistance of the *lox3-4* mutant (Figure 8).

Interestingly, both *ZmLOX5* and *ZmLOX10* were down-regulated in response to *C. graminicola* in leaves of WT plants treated with *lox3-4* sap, while in plants treated with WT sap, *ZmLOX5* expression was similar to control and *ZmLOX10* was up-regulated. Our previous work showed that both *lox5* (Park, 2011) and *lox10* (Christensen, 2009) mutants were more resistant to *C. graminicola*, suggesting that these two genes are susceptibility factors in maize. Suppression of these two genes in the *lox3-4-*sap treated plants is a logical explanation of yet another mechanism behind increased resistance in *lox3-4* mutant (Figure 8). The role of these genes in the *lox3* mutant background is currently under investigation through generation of double mutants in our laboratory.

Previous analyses showed that JA, ET, SA, and genes involved in JA biosynthesis pathway including *LOX8*, *AOS*, *AOC*, and ethylene biosynthesis gene *ACO31*, are up-regulated in *lox3-4* maize roots (Gao et al., 2007, 2008). These findings suggest that JA and/or ethylene may themselves be mobile signals present in the *lox3-4* sap that activate systemic resistance. Alternatively, increased JA/ethylene levels result in transcriptional activation of other pathways that produce the systemic mobile signal (Figure 8).

H_2_O_2_ and NO are synergistic in their signaling of defense responses, especially to biotrophs and hemibiotrophs (Mittler et al., 2004; Besson-Bard et al., 2008). Earlier and higher accumulation of H_2_O_2_ and NO in response to *C. graminicola* and/or ALA treatment together with earlier induction and greater expression of diverse set of defense related genes, *PR1*, *PAL*, *GST*, and *MPI*, in *lox3-4* leaves may explain the increased resistance of this mutant. H_2_O_2_ was shown to limit growth of hemibiotroph *Septoria tritici* (Shetty et al., 2007) and the ability to scavenge and detoxify ROS is directly related to growth and virulence of hemibiotroph *M. oryzea* (Huang et al., 2011) and necrotroph *Sclerotinia sclerotiorum* (Veluchamy et al., 2012). In *C. graminicola* infected B73, H_2_O_2_ started to accumulate at ~36 hpi, which is similar to previous reports (Vargas et al., 2012). However, *lox3-4* mutants started to accumulate H_2_O_2_ earlier (~24 hpi). In addition to direct effect on fungal growth, H_2_O_2_ is associated with cell wall lignification and cross-linking of cell wall proteins that further limits fungal growth. Continued increase in H_2_O_2_ when fungi enter the necrotrophic stage is suggested to contribute to plant cell death and disease development. *lox3-4* leaves continued to accumulate H_2_O_2_ even at ~72 hpi, during the necrotrophic stage. Significant increase of *GST* expression in the leaves of mutant plants compared to wild type may help explain the lesser extent of necrotrophic lesion in these plants compared to WT, since GST is associated with quantitative disease resistance in maize and is implicated in resistance to multiple maize diseases (Wisser et al., 2011).

A recent study has implicated an *Arabidopsis* 9-lipoxygenase, AtLOX5, and its 9-oxylipins in susceptibility to foliar green peach aphid (GPA) (Nalam et al., 2012), and showed that the movement of GPA inducible 9-oxylipins via xylem sap facilitate GPA growth and host colonization. These studies and our previous reports (Gao et al., 2007; Christensen, 2009; Park, 2011) identified *AtLOX5*, *ZmLOX3, ZmLOX5*, and *ZmLOX10* as “susceptibility factors” and possible targets for pathogen or insects manipulation. This raises the question of why these genes have perpetuated throughout plant evolution. The unprecedented observation that *ZmLOX3* is downregulated by a beneficial root-colonizing fungus provides a possible explanation. Ubiquitous *Trichoderma* spp. with a wide host range may naturally downregulate this and/or other LOX genes in the host leading to de-repression/activation of ISR only when needed. The ecological cost of expressing secondary defense mechanisms such as SAR and ISR justifies the presence of their negative regulators at one or multiple layers, whose down-regulation in response to environmental cues allows for initiation and activation of the secondary defense processes. Besides *Trichoderma* spp., other ISR inducing microorganisms interact with maize roots in the rhizosphere and it is yet to be seen whether suppression of 9-LOXs by mutualistic rhizosphere microbes such as plant growth promoting rhizobacteria is conserved. Whether this gene is permanently suppressed in colonized plants remains to be determined.

## Conflict of interest statement

The authors declare that the research was conducted in the absence of any commercial or financial relationships that could be construed as a potential conflict of interest.

## Supplementary material

Supplemental Figure 1 **(A)** Image depicting hydroponic system. Setup allows for plant roots to be submerged in liquid media allowing root inoculation. **(B)** Image depicts xylem sap collected from V4 maize plants forming as droplets at cut sites. Cuts were made at a diagonal angle with a razor blade. **(C)** Illustrated image depicting dimensions of incision sites.

Supplementary Table 1 **Primers used in this study for RT-PCR and Q-RT-PCR**.
