# Supplementary figures and images for: Root-expressed maize lipoxygenase 3 negatively regulates induced systemic resistance to Colletotrichum graminicola in shoots
Source: Front Plant Sci. 2013 Dec 18;4:510. doi: 10.3389/fpls.2013.00510 (PMC3867115; doi:10.3389/fpls.2013.00510)

A.

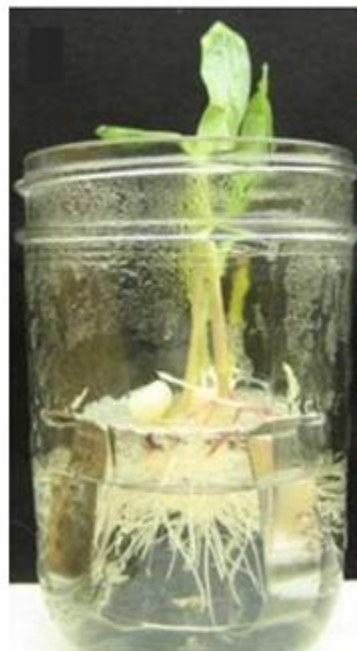

B.

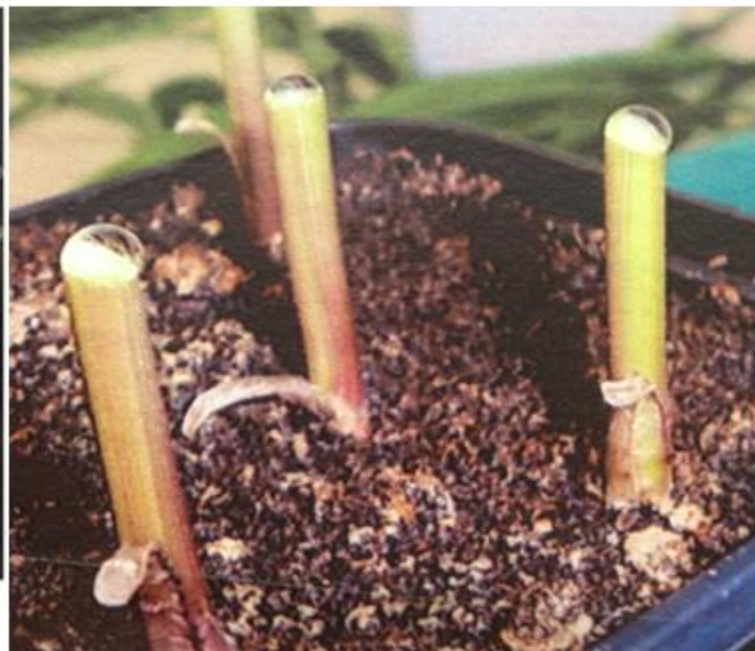

C.

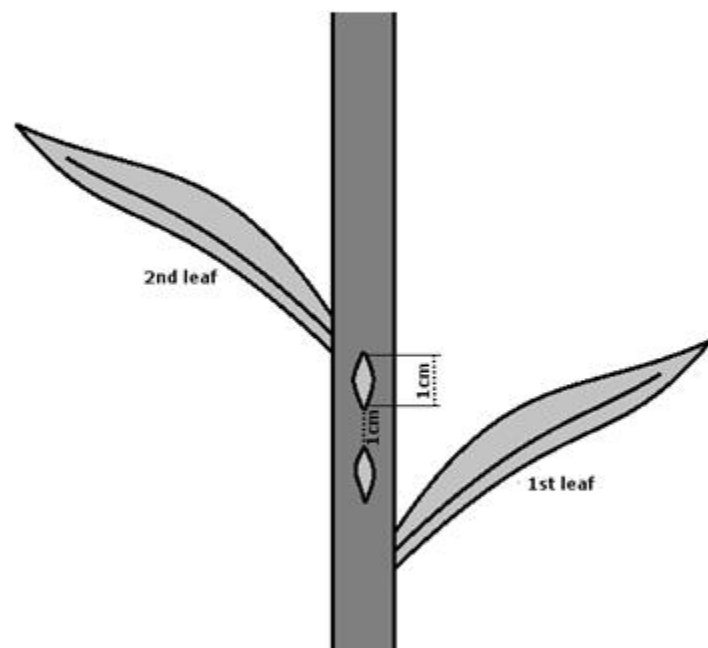

Supplement: Supplemental Figure 1 — (A) Image depicting hydroponic system. Setup allows for plant roots to be submerged in liquid media allowing root inoculation. (B) Image depicts xylem sap collected from V4 maize plants forming as droplets at cut sites. Cuts were made at a diagonal angle with a razor blade. (C) Illustrated image depicting dimensions of incision sites. [file Presentation1.PDF]
